# Empathy Increased in Rural and Remote Health and Social Care Workers by Participation in the Hearing Voices That Are Distressing Simulation Workshop

**DOI:** 10.1111/inm.70027

**Published:** 2025-03-18

**Authors:** Carol‐Ann Stanborough, Chloe M. E. Fletcher, Lee Martinez

**Affiliations:** ^1^ Department of Rural Health, Rural & Remote Health SA College of Medicine and Public Health, Flinders University Renmark South Australia Australia; ^2^ IIMPACT in Health, Department of Rural Health, Allied Health and Human Performance University of South Australia Adelaide South Australia Australia

**Keywords:** empathy, hearing voices, mental health, rural, simulation education

## Abstract

Evidence suggests that health care workers are often uncomfortable talking with people about hearing voices, despite recommendations that voice‐hearers be provided with opportunities to freely discuss their experiences. Moreover, in rural and remote Australia, workforce shortages mean that a broader range of workers, often non‐specialists, are providing services for people with complex mental health presentations. Improving the skills of this non‐specialist workforce is therefore an important endeavour. The Hearing Voices that are Distressing (HVD) simulation workshop was originally developed by voice‐hearers and provides participants with first‐hand experience of what it might be like to hear voices that are distressing. HVD simulation workshops were delivered by a mental health academic, a mental health clinician, and a person with lived experience to 62 health and social care workers in rural South Australia. Mixed methods were used to examine the impact of the workshop on participants' level of empathy for people who hear distressing voices. The revised Kiersma‐Chen Empathy Scale assessed changes in empathy, and post‐simulation reflective group discussions were qualitatively analysed. Statistically significant increases in empathy were reported following participation in the simulation (p's < 0.001). Participants reflected that having practical experience helped them develop deeper understanding of the impact hearing distressing voices has on a person's day‐to‐day life; how they may be preoccupied with their internal world; impacting their concentration and engagement with others. Results indicated that this training would be highly valuable for health and social care workers, and particularly generalist workers in rural settings where resources are stretched.

## 1 Introduction

When mental health care providers have insight into a person's lived or living experience it creates connection, supporting the delivery of therapeutic care and ultimately the person's journey towards recovery. Key findings within mental health lived experience research have shown that people with lived or living experience, and their families and carers, want to receive care from health professionals whom they can trust, who listen with empathy and non‐judgement, who have insight and are non‐stigmatising in their approach (Kaine and Lawn 2021).

Empathy is a core component of a person‐centred approach and is described as the ability to understand and share the feelings of another person. It involves putting oneself in someone else's shoes, seeing things from their perspective, and experiencing the world as they do. Having empathy also involves being able to recognise and validate the emotions of others without judgement. To do this requires active listening, emotional intelligence, and a willingness to be present and attuned to someone else's experiences (Riess 2017). Empathy is a foundational skill when working in health and social care settings, as Carl Rogers suggested, “…empathy is in itself a healing agent. … because it releases, it confirms, it brings even the most frightened client into the human race. If a person can be understood, he or she belongs” (Rogers 1986, 129).

Recent advances in the field of neurobiology have shed light on the underlying mechanisms of empathy, revealing that it is not just a ‘soft skill’ but rather a neurobiologically based competency (Riess 2010). Specifically, research has demonstrated that when we empathise with others, our brains simulate similar emotional and sensory experiences, allowing us to understand and resonate with another person's feelings.

From a therapeutic perspective, Riess (2017) asserts that empathy is fundamental to establishing a trusting therapeutic relationship. Recent research highlights the importance of therapeutic trust, particularly when supporting people living with severe and enduring mental health conditions or complex trauma (Chouliara et al. 2023). When trust is established, the person receiving therapeutic support is more likely to take on advice, try out recommended treatment plans, and adhere to medication, ultimately contributing to a greater likelihood of recovery.

Hearing voices (auditory hallucinations) is generally considered to be indicative of schizophrenia spectrum or other psychotic disorders. Despite research suggesting that it is relatively common (Beavan et al. 2011; Toh et al. 2022), the experience of hearing voices remains highly stigmatised (Colizzi et al. 2020; Reavley et al. 2014). Consequently, people who hear voices can be reluctant to talk about their experiences (de Jager et al. 2016), and this can lead to social isolation, alienation (Vilhauer 2017) and avoidance of seeking help when needed (Bogen‐Johnston et al. 2019). In this context, it is paramount that when voice‐hearers do disclose their experience, they are met with empathy and understanding.

Interventions designed to enhance empathy are often used at an undergraduate level to support students' clinical development. In their systematic review examining the effectiveness of empathy interventions, Levett‐Jones et al. (2019) found those that were most effective focused on vulnerable patient groups, involved immersive and experiential simulations, and included opportunities for guided reflection. The ‘Hearing Voices that are Distressing’ (HVD) simulation workshops incorporate each of these aspects, allowing mental health and social care workers, clinicians, and students to gain an understanding of the challenges that people who hear distressing voices face daily. The HVD simulation workshop is an adaption by Arana Pearson based on Patricia Deegan's groundbreaking work in this space (Deegan 2024). The workshop aspires to the principles of the Hearing Voices Network (HVN), which originated in the Netherlands in the 1980s and contributed to a profound shift away from dominant perspectives that ‘…voices are meaningless hallucinations, signifying severe mental illness, and that only through silencing and blocking them out could people be returned to good mental health’ (Hearing Voices Network SA n.d.). Instead, voice‐hearing is approached from a place of curiosity, with the understanding that it is a common human experience, often linked to trauma, and may carry important information about the voice‐hearer's past or present experiences (InterVoice n.d.).

In 2015, the University of South Australia (UniSA) Department of Rural Health engaged Arana Pearson to train academics, mental health clinicians, and people with a lived experience of voice‐hearing to deliver the HVD simulation workshop. Since this time, the workshop has been delivered across regional and rural South Australia, co‐facilitated by a person with lived experience of hearing distressing voices and a clinician and/or a rural mental health academic. In 2018, we conducted preliminary evaluation research examining the benefits of the HVD simulation workshops for rural students, students on rural clinical placement, and health workers (Martinez and Walker‐Jeffreys, unpubl. data 2018). Our findings indicated a significant shift in empathy among that cohort. The present study builds on that work, aiming to increase our understanding of whether participation in the HVD simulation workshop leads to an increase in empathy for people who hear distressing voices among rural health and social care workers and to provide insight as to how this change occurs.

The use of voice hearing simulation in education was first implemented by Deegan in 2006. In the subsequent decade and a half, there remains a small number of publications reporting the impact and benefits to learning that this education may provide, and these predominantly involve nursing students in the USA and Canada (Chaffin and Adams 2013; Dearing and Steadman 2009; Kim and Wojnar 2019). Combined, these findings indicate that the simulation provides an impactful and unique learning experience that allows students to walk in voice‐hearers' shoes, thereby increasing their empathy and understanding (Chaffin and Adams 2013; Dearing and Steadman 2009; Kim and Wojnar 2019). In Australia, research in this area is emerging, with two studies located; one by Orr et al. (2013) with nursing students and one by Patterson et al. (2014) with multidisciplinary professionals based in a large metropolitan hospital. To our knowledge, the present study is the only one involving a broader cohort of participants who are health, mental health, and social care workers—both professional and non‐professional—who are based in rural Australia.

## 2 Method

### 2.1 Study Design

A mixed methods study design was used to evaluate the HVD simulation workshop. Our purpose in using a mixed methods study design was to quantitatively examine whether participation in the workshop led to an increase in empathy for people who hear distressing voices and to qualitatively explore how the simulation experience may have contributed to this change. In terms of quantitative methods, a modified version of the revised Kiersma‐Chen Empathy Scale (KCES‐R) (Aronson et al. 2022) was used to evaluate the impact of the workshop on participants' self‐reported empathy. Participants completed the quantitative measure at two time points: at the beginning of the workshop (i.e., prior to the theoretical lecture) and at the end of the workshop (i.e., following the debriefing and reflective session). To provide insight as to how a change in empathy may occur, the reflections participants shared during the structured debriefing and reflective session were audio recorded and analysed using qualitative methods.

### 2.2 Participants

Participants were health and social care workers in rural South Australia who worked with people experiencing mental health challenges (including hearing distressing voices). These included primary care workers in community health, regional health services (including public hospitals), and non‐government organisations. To be eligible, participants needed to have enrolled in the workshop and be aged 18 years or older. The first and senior authors' extensive professional networks were utilised to advertise workshops within rural health and social care services, including community health services, public hospitals, and non‐government agencies. Emails were sent to managers, leaders, and administration staff with information about the workshop being delivered in their area, and a flyer was provided to distribute among staff.

### 2.3 Settings

Five workshops were presented in four rural and remote locations in South Australia: Murray Bridge, Berri, Port Augusta (two workshops), and Whyalla. The Modified Monash (MM) model classifies Australian locations based on their geographical remoteness. Categories range from MM 1 (metropolitan areas) to MM 7 (very remote communities). The model is commonly used to understand the geographical distribution of the health workforce and address disparities in access to healthcare. Locations where HVD workshops were held range from small (MM 5; Berri) to medium (MM 4; Port Augusta) and large rural towns (MM3; Murray Bridge and Whyalla).

### 2.4 HVD Simulation Workshops

Workshops were run by rural mental health academics from UniSA Department of Rural Health (LM and C‐AS) in partnership with a lived experience facilitator (MM or FW) and were delivered over 3.5–4 h, divided into 4 parts: (1) a video introduction from Arana Pearson sharing his own story of voice‐hearing, (2) a 45‐min theoretical lecture on current research and techniques that can be used to give voice‐hearers power over their voices, with time dedicated to the LE facilitator to share his experiences, (3) a 35–45‐min simulation experience, and (4) a debrief group discussion for 30–40 min. During the simulation, participants listen to a recording of distressing voices via an MP3 player while engaging in activities that one might be expected to participate in while attending an inpatient or community mental health service (e.g., undertaking a mental state assessment, participating in a group activity, engaging in an everyday activity, such as asking for directions or ordering a coffee). The recording includes sounds, words, and conversations in different tones and volumes, as well as periods of silence, taking participants on a journey of what it might be like to hear distressing voices. The simulation is followed by a debrief session for participants to re‐ground in the present moment, share their experiences, and consolidate their learnings from the workshop. Throughout the workshop and during the debrief session, participants engage in discussion and are encouraged to ask the ‘difficult‐to‐ask’ questions.

### 2.5 Evaluation

#### 2.5.1 Procedure

HVD simulation workshops were held between 20 June and 19 July 2022.

Ethical approval was obtained from the Human Research Ethics Committee of the University of South Australia (Protocol 204 496). Participation in the study was voluntary, and participants could withdraw at any stage. Participants signed a consent form to be part of the study, but those who declined involvement in the study were still able to participate in the workshop itself.

Participants completed quantitative measures at the beginning and end of the workshops. Paper‐copy surveys were given to participants by C‐AS at each time point. Participants took 5–10 min to complete the survey. C‐AS collected completed surveys and entered data into a Microsoft Excel spreadsheet prior to analysis. Structured debriefing and reflective sessions were facilitated by LM, C‐AS, and MM at the end of each workshop and generally lasted 30–40 min. All sessions were audio‐recorded and transcribed in preparation for qualitative analysis. Field notes were taken by both LM and C‐AS.

#### 2.5.2 Quantitative Survey

Participants completed a modified version of the KCES‐R (Aronson et al. 2022) at the beginning and end of each workshop. The KCES‐R was used instead of the more commonly used Jefferson Scale of Empathy (Hojat et al. 2018) because it is open‐access, free to use, and simple to administer. The scale completed by participants is available in Supporting Information.

The KCES‐R measures two distinct domains of cognitive and affective empathy. The cognitive domain assesses individuals' ability to understand and view the world from other people's perspectives, whereas the affective domain assesses individuals' ability to connect to the experiences or feelings of others. The questionnaire is split into two sections, the first measuring perceptions of the importance of empathy among healthcare professionals generally, and the second measuring self‐perceptions of empathy. Each section has seven items with parallel statements, including seven general statements (e.g., It is necessary for healthcare professionals to comprehend voice‐hearers' experiences) and seven similarly worded self‐perception statements (e.g., I am able to identify with voice‐hearers' feelings). General statements were rated using a seven‐point Likert scale from 1 = Unnecessary to 7 = Extremely necessary and self‐perception statements were rated from 1 = Does not describe me to 7 = Describes me extremely well. In the present study, the internal consistency of the cognitive and affective subscales was assessed using Cronbach's alpha (0.702 and 0.797, respectively).

Demographic information (e.g., gender, age, employment, education) was collected as part of the baseline survey to describe the study participants.

#### 2.5.3 Structured Debriefing and Reflective Sessions

Structured debriefing and reflective sessions were facilitated at the end of each workshop and were audio‐recorded. Five open‐ended questions were asked during the reflective sessions: (a) How did participation in the workshop contribute to your understanding of the experiences of a person who hears voices? (b) What was your key learning from the content of the workshop? (c) What did you like most about the workshop? (d) What did you find least helpful about the workshop? and (e) How do you think participation in the workshop may change your health practice? All sessions were audio‐recorded, and field notes were taken by both LM and C‐AS.

#### 2.5.4 Data Analysis

Quantitative data analysis was performed by KM and CMEF using Microsoft Excel and IBM SPSS Statistics (version 28). Sixty‐two participants attended the workshops; however, one participant did not complete the survey at the end of the workshop (response rate = 98%). Because of this, only data from 61 participants were included in the analysis. Descriptive statistics were used to analyse the demographic data. Consistent with similar earlier studies (e.g., Chaffin and Adams 2013), paired samples t‐tests were conducted to evaluate the impact of participation in the HVD simulation on self‐reported empathy (measured using the KCES‐R). The Kolmogorov–Smirnov test was conducted to examine the distribution of scores on the cognitive and affective subscales of the KCES‐R. Pre‐workshop scores on the cognitive scale (*p* = 0.002) and pre‐ and post‐workshop scores on the affective scale (*p* = 0.004 and < 0.001, respectively) were found to be non‐normally distributed. Hence, nonparametric tests were performed to confirm the findings of paired samples *t*‐tests.

Qualitative data analysis was conducted by CMEF, a post‐doctoral research fellow and community mental health practitioner, who works with people who hear voices. It is important to acknowledge that CMEF also participated in the HVD simulation as an experiential learning experience and therefore is positioned as both a participant and researcher. Acknowledging CMEF's dual role as a participant and researcher, the analysis was underpinned by an interpretivist epistemology. The interpretivist position assumes that different people experience and understand the same ‘objective reality’ in different ways, including all research participants and the researchers conducting the analysis. This epistemology fitted our research aim to explore participants' experiences of the workshop, including the theoretical lecture and simulation components. Therefore, it was assumed that participants experienced the workshop and made sense of their experiences differently, and that their experiences were interpreted through the lens of the researcher's own experience.

Transcripts from the debriefing and reflective sessions were subjected to reflexive thematic analysis using an inductive approach and guided by Braun and Clarke's (2006) six phase process. Transcripts were imported into NVivo 12, which was used to code and organise the data. In phase one of the analysis process, CMEF became familiar with the data by reading the transcripts while listening to corresponding audio recordings. Throughout this process, CMEF made notes on patterns and meanings that were observed within the data. In phase two, CMEF read the transcripts again and began to generate initial codes (short phrases) to label data items at the semantic (descriptive) level (e.g., ‘hearing from lived experience was valuable’). In phase three, CMEF reviewed the codes and identified relationships between codes. These were sorted into initial themes, comprised of related codes centred around a shared meaning. In phase four, CMEF returned to the data to review and refine codes and themes, to ensure that they described a coherent narrative that captured the essence of participants' experiences. In phase five, CMEF prepared a preliminary report outlining the definition and name of each theme. This was reviewed by LM and C‐AS in phase six.

## 3 Results

### 3.1 Participant Demographics

Sixty‐one participants completed both questionnaires. Participants were predominantly female (77%) with an average age of 42 years (range = 20–67 years). Participants were employed within the mental health sector and government (71%) and non‐government (24%) sub‐sectors, primarily in roles in nursing (23%), mental health (19%), support work (18%), and social work (15%). Participant characteristics are described in Table 1.

**TABLE 1:** Participant characteristics.

Characteristic	*M* (range)	Number	Percentage (%)
Age (years)	42 (20–67)		
Gender			
Female		47	77
Male		14	23
Employment status			
Government agency		44	71
Non‐government organisation		15	24
University		2	3
Unemployed		1	2
Length of current employment (years)	3.27 (< 1 to 20)		
Highest educational qualification completed			
High school		8	13
Certificate or diploma		16	26
Undergraduate degree		12	20
Postgraduate degree, diploma, or certificate		24	39
Missing		1	2
Current role			
Mental health worker		12	19
Registered nurse		11	18
Support worker		10	18
Social worker		9	15
Manager		2	5
Enrolled nurse		3	5
Psychologist		2	3
Occupational therapist		2	3
Service coordinator		2	3
Administrator		2	3
Assistant allied health worker		1	2
Academic		1	2
Student		2	3
Unemployed		1	2

Abbreviation: *M*, mean.

### 3.2 Change in Self‐Reported Empathy Following Participation in the Simulation

Paired samples t‐tests revealed statistically significant increases in both cognitive and affective empathy after participation in the simulation (see Table 2). Because data were non‐normally distributed, Wilcoxon signed‐rank tests were conducted to confirm the findings of paired sample t‐tests. Wilcoxon signed‐rank tests confirmed statistically significant increases in both cognitive (*Z* = −6.465, *p* = < 0.001) and affective (*Z* = −6.416, *p* = < 0.001) empathy.

**TABLE 2:** Change in composite scores on cognitive and affective empathy scales following participation in the HVD simulation.

	Pre‐simulation, *M* (SD)	Post‐simulation, *M* (SD)	Change, *M* (SD)	*t*	df	*p*	Cohen's *d*
Cognitive empathy	39.62 (4.62)	46.10 (2.52)	6.48 (4.22)	−11.899	59	< 0.001	−1.536
Affective empathy	40.18 (5.41)	46.54 (2.71)	6.36 (4.69)	−10.599	60	< 0.001	−1.357

*Note:* *p* < 0.05 indicates statistical significance.

Abbreviations: *d*, effect size; df, degrees of freedom; M, mean; *p*, *p*‐value; SD, standard deviation; *t*, *t*‐statistic.

Wilcoxon signed‐rank tests revealed statistically significant increases in individual item scores following participation in the simulation (see Table 3) for all items, except items 3 (It is necessary for healthcare professionals to be able to value voice hearers' point of view) and 6 (It is necessary for healthcare professionals to be able to identify with voice hearers' feelings) (*p* = 0.059 and 0.060, respectively).

**TABLE 3:** Change in item scores following participation in the HVD simulation.

KCES‐R item	Pre‐simulation, *M* (SD)	Post‐simulation, *M* (SD)	*Z*	*p*
**Section 1: It is necessary for healthcare professionals to be able to…** ^a^				
Comprehend voice hearers' experiences.	6.53 (0.79)	6.87 (0.34)	−3.463	< 0.001
2 Express an understanding of voice hearers' feelings.	6.56 (0.85)	6.87 (0.34)	−3.082	0.002
3 Value voice hearers' point of view.	6.77 (0.59)	6.90 (0.30)	−1.890	0.059
4 Consider voice hearers' feelings to provide consumer‐centred care.	6.77 (0.62)	6.92 (0.28)	−2.310	0.021
5 Be caring in order to build a strong relationship with voice hearers.	6.82 (0.53)	6.93 (0.25)	−2.111	0.035
6 Identify with voice hearers' feelings.	6.49 (0.89)	6.64 (0.97)	−1.881	0.060
7 View the world from the voice hearers' perspective.	6.46 (0.83)	6.80 (0.44)	−3.586	< 0.001
**Section 2: I am able to…** ^b^				
8 Comprehend voice hearers' experiences.	3.97 (1.46)	6.13 (0.81)	−6.268	< 0.001
9 Express an understanding of voice hearers' feelings.	4.36 (1.48)	6.29 (0.76)	−6.186	< 0.001
10 Value voice hearers' point of view.	5.25 (1.42)	6.51 (0.67)	−5.483	< 0.001
11 Consider voice hearers' feelings to provide consumer‐centred care.	5.28 (1.39)	6.54 (0.65)	−5.486	< 0.001
12 Be caring in order to build a strong relationship with voice hearers.	5.79 (1.27)	6.64 (0.63)	−4.792	< 0.001
13 Identify with voice hearers' feelings.	4.61 (1.58)	6.34 (0.79)	−6.096	< 0.001
14 View the world from the voice hearers' perspective.	4.20 (1.66)	6.29 (0.80)	−6.112	< 0.001

*Note:* *p* < 0.05 indicates statistical significance.

Abbreviations: *M*, mean; *p*, *p*‐value; SD, standard deviation; *Z*, *Z*‐score.

^a^ Perceptions of the importance of empathy among healthcare professionals generally. Response scale: 1–7 (1 = Unnecessary, 4 = Moderately necessary, 7 = Extremely necessary).

^b^ Self‐perceptions of empathy. Response scale: 1–7 (1 = Does not describe me, 4 = Describes me moderately well, 7 = Describes me extremely well).

### 3.3 Participants' Reflections on the Simulation Experience

The reflections that participants shared helped to explain how the simulation experience may have contributed to an increase in empathy for people who hear distressing voices. Six sub‐themes were developed through qualitative analysis of the reflective sessions, though there was an overarching theme that emphasised the value of having practical experience for gaining insight into voice‐hearers' experiences. Sub‐themes explored different aspects of insight that participants gained to voice‐hearers' experiences. A summary of themes with illustrative quotes is presented in Table 4.

**TABLE 4:** Summary of themes generated from debrief sessions conducted with rural health and social care workers immediately following the simulation.

Themes	Illustrative quotes
*Over‐arching theme*	
Having practical experience was valuable for gaining insight to voice‐hearers' experiences	“… actually, experiencing that for 25 min—how terrifying, or distressing – like really scary, just yeah it was something that I will sort of carry with me…” (female participant, Port Augusta PM workshop) “… it has given me like a broad and deeper understanding of, of what clients go through with those voices. And it will change like my way of delivery of the services and looking at the client as a whole and be more empathetic and making them feel comfortable, supported and connected with the services.” (male participant, Berri workshop)
*Sub‐themes*	
“…all I wanted to do was switch it off”: Insight to how it feels to hear distressing voices	*“*I felt really uneasy because I just didn't know what was going on. I couldn't—I generally need to know what's going on—and I couldn't know what was going on. And as time went by, I just felt myself shutdown, shutdown response sort of thing and just don't want to be part of it.” (female participant, Berri workshop) “Having that snapshot … all I wanted to do was switch it off. So, understanding that they [the voices] can't be switched off is quite shocking…” (female participant, Port Augusta AM workshop) “I knew I was going to take them off, and I was going to walk away. And the bit that doesn't sit well is… [a voice‐hearer] doesn't have that [choice].” (male participant, Port Augusta PM workshop) “For us it's kind of an interesting, a little bit funny and we knew that we are doing some activity and not really going through those. So, it gives us many insights about our clients who are really going through this. Must be very difficult for them.” (male participant, Berri workshop)
2 “…in everyday stuff, it would be very debilitating”: Insight to difficulties voice‐hearers experience during conversation and in concentration, comprehension, and information recall	“…when the [role‐played] doctor was asking questions, I couldn't hear half of it, when you were giving your coffee order [as part of the activity], I couldn't hear what you were saying or what I thought you said; just in everyday stuff, it would be very debilitating.” (female participant, Port Augusta AM workshop) “I … could not, I could not concentrate, just generally, even just general reading it would not sync in my head. The thoughts were there are just, God, this is driving me crazy, basically, it was terrible.” (female participant, Berri workshop) “… you had to concentrate more too, to listen to people … so I found I was concentrating more which probably if you've got to do that all day every day it's pretty exhausting.” (female participant, Port Augusta AM workshop) “I think my take home is just how exhausting it was. Trying to do the reading exercise, and I literally, I just could not do that.” (female participant, Murray Bridge workshop) “I was doubting my memory there, what you told me, my recollection, I was just doubting myself there.” (male participant, Whyalla workshop) “I know the answers to the mini‐mental. I memorised this, but I could not recall.” (female participant, Murray Bridge workshop)
3 “I had to stop myself responding and saying shut up”: Insight to behaviours voice‐hearers might display	“I felt that I couldn't even talk to you properly when I was relaying the order back to you, because the voices were so loud that I had to be so much louder.” (female participant, Port Augusta PM workshop) “And the sound in your ears was louder than everyone talking too. So, and I was always, like it crossed my mind, I was actually saying, was I yelling, I just felt, that am I actually speaking louder than I normally do?” (male participant, Berri workshop) “I felt that I was probably perceived quite aggressive, because of my tone.” (female participant, Port Augusta PM workshop) “…if you put yourself on the other side of the fence and someone's watching me and all of a sudden, I'm jumping around or I'm laughing or I'm looking at something and it's—well obviously you're psychotic.” (female participant, Port Augusta AM workshop) “I had to stop myself responding and saying shut up.” (female participant, Berri workshop) “…the [voices were] laughing, and laughing can be quite, what's the word, contagious and I found myself having a bit of a laugh.” (female participant, Port Augusta AM workshop)
4 “…how do we challenge colleagues respectfully to change perspectives?”: Insight to role health and social care workers can play in challenging stigma and providing a safe environment for voice‐hearers	“I was thinking about what our role is and what I could offer in terms of challenging other people in terms of their belief systems, bias systems. Because I think when it's a lack of education in the community and among colleagues as well, sometimes we have to acknowledge how challenging it is to work with people. And what that can bring out in us. And I think if that is bringing something out in us, and we're not responding like we should, … what's that showing to other people. And so, calling people out if they're saying those people are ‘crazy’ … I think it's taking that broader, that individual interaction but how to challenge community, how do we challenge colleagues respectfully to change perspectives, I think that's a big core thing.” (female participant, Berri workshop)
5 “…is this what we do to people?”: Insight to how current processes and systems can harm voice‐hearers	“I wonder if, as mental health professionals, we just go too fast and we fire too many things at once because we're under time pressure or whatever else, we've got our own tasks to get done. Because that's what I couldn't stand was everything was being fired at us at a thousand times a second and there was not a chance to ask a question or clarify or anything else like that. So that was what I was thinking was, is this what we do to people? … A sentence was begun looking at us, and then they walked away from us and I'm thinking I can't hear the rest of that sentence because of everything else. We think we're communicating well but are we really? I'm not so sure about that.” (female participant, Murray Bridge workshop) “I was a little upset because I couldn't answer the [role‐played] doctor. I could hear him talking to me and looking at me and because I couldn't answer, and he says do you want to go to hospital. And then I said no. And I'm thinking is that something that we ask our clients when they don't respond? We think they're unwell but—and I can vaguely remember I could hear that. That was about all I could hear. And if that was a real scenario, how overwhelming.” (female participant, Murray Bridge workshop) “And I felt very invalidated because I'm obviously presenting in—in distress. And the [role‐played] doctor didn't pick up—you know, I felt he should've asked, you know, are you okay? Are you—you feel a bit distressed? Like no, I felt—I felt really invalidated, yeah.” (male participant, Port Augusta PM workshop) “It's the environment, put them in ED in a cubicle. … They can be down there for three days or more in a tiny cubicle, noisy, bright and it can just be like—I was doing joint care with a few of them just to try and keep patients who are waiting for admissions settled and it can be—the general nurses just don't want to deal with it often. Can I have a coffee or a sandwich or something? Bored probably, but that's fine. Just go and get it for them. They won't, so they escalate, you get a code black.” (male participant, Whyalla workshop)
6 “…taking a step back and thinking a little bit more and exploring”: Insight to how practice can better meet voice‐hearers' needs	“I think really getting an understanding of what the experience is … What does [hearing voices] mean to them? And where is it coming from and is it causing distress and is it impairing function?” (female participant, Murray Bridge workshop) “My main learning is probably just similar to everyone else, being more explorative and curious with people that might be hearing voices. And probably just thinking about the basis of voices as well, like whether it was cultural or spiritual, like for Aboriginal people and explore it more.” (female participant, Port Augusta PM workshop)

#### 3.3.1 Over‐Arching Theme: Having Practical Experience Was Valuable For Gaining Insight to Voice‐Hearers' Experiences

Throughout the debrief sessions, it was clear that participating in the simulation provided health and social care workers with insight into what it might be like to hear distressing voices. Participants discussed that the simulation provided insight into what voices might say (e.g., derogatory statements) and how this might impact voice‐hearers (e.g., reduce confidence) and their ability to engage with their external environment (e.g., in conversation).

Participants also gained insight into what it might be like to engage with mental health professionals and health care systems as a voice‐hearer. They reflected on the stigma that voice‐hearers might experience (e.g., judgement from others) and reasons they might be reluctant to engage with mainstream services (e.g., services not designed with consideration for voice‐hearers' unique needs). The following sub‐themes explore participants' reflections in more detail.

#### 3.3.2 Sub‐Theme 1: “…all I wanted to do was switch it off”: Insight to How It Feels to Hear Distressing Voices

Participants described strong negative reactions to the simulation, including hypervigilance, dissociation, and feelings of wanting to escape. They said they felt uneasy, distressed, frustrated, and distracted during the simulation and described the voices as exhausting, unpredictable, and isolating. One participant said:

…experiencing that for 25 min – how terrifying, or distressing – like really scary, yeah it was something that I will carry with me. (female participant, Port Augusta PM workshop)

Another participant described a sense of losing control, leading to a shutdown response:

I felt really uneasy because I just didn't know what was going on. … as time went by, I just felt myself shutdown … shutdown response sort of thing and just don't want to be part of it. (female participant, Berri workshop)

Some participants described their uncomfortable realisation that although the simulation was a temporary experience for them, it was not a temporary experience for people who live with distressing voices. This helped to put the experience into perspective, potentially deepening their empathic response.

#### 3.3.3 Sub‐Theme 2: “…in everyday stuff, it would be very debilitating”: Insight to Difficulties Voice‐Hearers Experience During Conversation and in Concentration, Comprehension, and Information Recall

Participants discussed how hearing distressing voices (on the recording) impacted their ability to engage in the simulation activities. Listening to the recording made it difficult for them to engage in conversation, to comprehend what others were saying or what they were reading, to respond to questions or recall information from memory, and to concentrate on the simulation activities. The additional focus and energy required to engage in the simulation activities left participants with an appreciation for how tiring it might be to live with distressing voices. One participant reflected on the simulation experience:

… you had to concentrate more too, to listen to people … so I found I was concentrating more which probably if you've got to do that all day every day it's pretty exhausting. (female participant, Port Augusta AM workshop)

#### 3.3.4 Sub‐Theme 3: “I had to stop myself responding and saying shut up”: Insight to Behaviours Voice‐Hearers Might Display

Participants also reflected on how their behaviour changed while they were hearing distressing voices (on the recording), which helped them to gain an understanding of the behaviours they might have seen in the voice‐hearers they work with. Some participants described having difficulty modulating the volume of their own voice (e.g., talking too loudly) and others talked about wanting to respond to or laugh at their voices. This prompted them to reflect on how these behaviours might be perceived by other people (e.g., as aggressive or ‘psychotic’). For example:

…if you put yourself on the other side of the fence and someone's watching me and all of a sudden, I'm jumping around or I'm laughing or I'm looking at something and it's – well obviously you're psychotic. (female participant, Port Augusta AM workshop)

#### 3.3.5 Sub‐Theme 4: “…how do we challenge colleagues respectfully to change perspectives?”: Insight to the Role Health and social Care Workers Can Play in Challenging Stigma and Providing a Safe Environment for Voice‐Hearers

Participants discussed the ways that they could challenge stigma in their workplaces and provide a safe environment for voice‐hearers. They talked about their role in challenging other people's beliefs about voice‐hearing and changing the culture within their workplace. One participant described challenging stigma as a core part of their work:

… I was thinking about what our role is and what I could offer in terms of challenging other people in terms of their belief systems, bias systems. Because I think when it's a lack of education in the community and amongst colleagues as well, sometimes we have to acknowledge how challenging it is to work with people. And what that can bring out in us. And I think if that is bringing something out in us, and we're not responding like we should, … what's that showing to other people. And so, calling people out if they're saying those people are crazy. (female participant, Berri workshop)

Participants also shared various strategies for creating change and providing a safe environment for voice‐hearers. These included educating their communities and colleagues (e.g., about what it is like to hear voices), being empathic and non‐judgemental in their approach, and making changes to their workspaces to increase voice‐hearers' feelings of safety (e.g., by offering to have radio on for background noise).

#### 3.3.6 Sub‐Theme 5: “…is this what we do to people?”: Insight to How Current Processes and Systems Can Harm Voice‐Hearers

Participants reflected on how mainstream mental health services can harm voice‐hearers. Some described feeling invalidated or frustrated during the simulation and considered how voice‐hearers in the real world may feel when engaging with mainstream services. For example:

I wonder if, as mental health professionals, we just go too fast and we fire too many things at once because we're under time pressure or whatever else, we've got our own tasks to get done. Because that's what I couldn't stand … everything was being fired at us at a thousand times a second and there was not a chance to ask a question or clarify or anything else like that. So that was what I was thinking was, is this what we do to people? (female participant, Murray Bridge workshop)

When reflecting on their current workplaces, participants identified workload and time as significant barriers to engaging with voice‐hearers in a person‐centred way. They felt that because of these barriers, they tended to communicate with voice‐hearers in ways that did not necessarily meet their needs (e.g., talking too fast, and with too much information communicated in too little time). Participants also commented on the inappropriateness of noisy and bright environments in emergency departments for people experiencing a mental health crisis and questioned whether mainstream mental health services were even designed with voice‐hearers' needs in mind.

#### 3.3.7 Sub‐Theme 6: “…taking a step back and thinking a little bit more and exploring”: Insight to How Practice Can Better Meet Voice‐Hearers' Needs

Participants discussed how they could change their practice to better meet voice‐hearers' needs. They emphasised the importance of using a person‐centred approach that involved being empathic, curious, and exploring the voice‐hearers' experiences and meanings with them. One participant explained:

I think really getting an understanding of what the experience is … What does [hearing voices] mean to them? And where is it coming from and is it causing distress and is it impairing function? (female participant, Murray Bridge workshop)

They also talked about the need to be patient and slow down their approach (e.g., having smaller goals or setting simple tasks), which they suggested included being thoughtful and flexible in how they communicated with voice‐hearers (e.g., using alternative communication methods).

## 4 Discussion

The aim of this mixed methods study was twofold: (1) to examine whether participation in the HVD simulation workshop led to an increase in empathy for people who hear distressing voices, and (2) to explore what it was about the simulation that may have contributed to this change. Our quantitative findings showed that participation contributed to a significant increase in both cognitive and affective empathy (p's < 0.001), suggesting that participants were better able to comprehend and express an understanding of what it is like to live with distressing voices following participation in the workshop. Our qualitative findings provided further context and insight to how the simulation enabled participants to experience what daily life might be like for someone who hears distressing voices. Themes spoke to the emotional and behavioural responses that one might have when hearing distressing voices, as well as the challenges related to other people's responses (e.g., stigma) and the potential harms that may be done by mainstream mental health services and practices. It is insights such as these that may have facilitated the increase in empathy demonstrated by our quantitative findings.

Our findings are consistent with previous research examining the use of voice‐hearing simulation among diverse healthcare professionals and students (e.g., Bradshaw et al. 2021). Participants in previous quantitative studies have reported significant increases in their level of empathy following the voice‐hearing simulation (e.g., Baisden and Gray 2020; Bunn and Terpstra 2009; Chaffin and Adams 2013). Similar to our findings, participants in qualitative studies reported a deeper understanding of voice‐hearers' experiences (Baisden and Gray 2020; Bunn and Terpstra 2009; Chaffin and Adams 2013; Dearing and Steadman 2008, 2009; Orr et al. 2013; Outlaw and Rushing 2018) and more knowledge about how to engage with people who hear distressing voices (Chaffin and Adams 2013; Dearing and Steadman 2008; Orr et al. 2013; Outlaw and Rushing 2018).

Reflecting on their experience of the simulation, participants in the present study discussed their own practice and the role they play in creating environments where voice‐hearers can feel safe, including challenging stigma within their workplaces and broader communities. These reflections are significant, given that conditions commonly associated with voice‐hearing, such as schizophrenia spectrum and other psychotic disorders, remain highly stigmatised (Colizzi et al. 2020; Reavley et al. 2014) and tend to be associated with representations of violence, dangerousness, and incompetence (Angermeyer and Dietrich 2006). Mental health workers are thought to be one of the main sources of stigmatisation for schizophrenia (Valery and Prouteau 2020). Of particular concern, stigmatising attitudes from mental health workers can negatively impact the quality of care provided to voice‐hearers (Reddyhough et al. 2021a). In fact, a recent study found that stigma was a significant barrier preventing Australian psychologists from working with people experiencing psychosis (Rackemann et al. 2024). We did not examine stigma specifically in the present study, and further research may be warranted to explore the relationship between empathy and stigma.

Another key reflection from participants was the importance of being curious and talking with voice‐hearers about their voices and what they mean to them. This is an important finding considering research suggesting that health care professionals are often reluctant to talk with people about their voice‐hearing (Coffey and Hewitt 2008; White et al. 2019). Although promising, it remains to be seen whether the cognitive learnings reported here translate to a change in practice in the longer term. Given the importance of the therapeutic relationship for supporting an individual's recovery (Hewitt and Coffey 2005; Horvath et al. 2011; Shattock et al. 2018), further work is needed to determine how participants apply learnings from the workshop within their practice.

Our work also extends previous findings relating to the value of simulation‐based training by exploring the use of this training method among workers in rural communities. It is well known that people living in rural and remote Australia face challenges related to the availability and accessibility of effective and appropriate mental health care (Duggan et al. 2020; Lawrence‐Bourne et al. 2020; Perkins et al. 2019). Workforce shortages in the mental health and social care sector mean that a broader range of workers, often non‐specialists, are providing services for people with complex mental health presentations in rural communities (Byrne et al. 2017; Fitzpatrick et al. 2018; Grant et al. 2018; Muyambi et al. 2022; Raviola et al. 2019). Improving the skills of this non‐specialist workforce is an important endeavour, given rural people accessing mental health services already face increased vulnerability related to greater visibility, stigma, and dual relationships within their communities (Procter et al. 2015). It is for this reason that we deliberately included workers from across the sector who may interact with voice hearers in the context of their work, rather than limiting participants to those in traditional clinical roles in psychology, social work, or mental health nursing. Participants, therefore, included mental health support workers, occupational therapists, allied health workers, and administrative support staff.

Limitations of this study should be addressed in future research. Notably, our sample size was relatively small (*n* = 61), and a more rigorous evaluation should be carried out with a larger sample. Participants also reported high baseline levels of empathy, potentially representing a self‐selection bias, where those who chose to participate had existing high levels of empathy for voice‐hearers. Alternatively, this may be reflective of the characteristics of those who choose to work in the, health and social care sectors. It is important to acknowledge that the simulation was one aspect of the workshops, and it is likely that the simulation itself contributes only in part to participants' increase in empathy. The workshop blends theoretical content with lived experience stories and is co‐facilitated by a person with lived experience of distressing voices who shares their own story and invites questions from participants. Reddyhough et al. (2021b) suggest that blended approaches involving education and social contact between participants and people with lived experience can be an effective way to challenge stigma surrounding voice‐hearing. We believe this is likely also the case for building empathy, however further research is needed to determine the unique contribution of the lived experience co‐facilitator to participants' increase in empathy for voice‐hearers.

Our findings indicate a significant increase in empathy following participation in the HVD simulation workshop; we therefore suggest that there is value in offering the workshop to rural and remote, health and social care workers as part of their training or ongoing professional development. There is also potential to incorporate the workshop into the curriculum for undergraduate psychology, medicine, nursing, and allied health students, with a particular focus on those who are interested in working rurally. Future research may also explore the adaptation of the HVD simulation workshop for family members, informal carers, and friends of people who hear distressing voices. Our next step is to conduct a pragmatic clinical trial to determine the unique contribution of lived experience co‐facilitation to building empathy for voice‐hearers through participation in the HVD simulation workshop. Through this work, we hope to expand the evidence base for the use of lived experience to enhance simulation‐based education and training for health and social care workers and students.

### 4.1 Conclusion

Empathy is described as the ability to understand and connect to the experiences and feelings of another person. It is a core component of a person‐centred approach and is shown to contribute significantly to positive experiences and outcomes for people accessing mental health care systems (Moudatsou et al. 2020; Nembhard et al. 2023). The HVD simulation workshop offers a unique opportunity for health and social care workers to gain an understanding of the challenges voice‐hearers face, and promisingly, our quantitative findings indicate that participation does contribute to an increase in empathy for people who hear distressing voices. This may be in part because the simulation provides participants with a brief glimpse into voice‐hearers' lives, as demonstrated by our qualitative findings which describe insights into the emotional and behavioural challenges that voice‐hearers may face internally (e.g., difficulties with concentration) and externally (e.g., stigma) in their daily lives. Our next step is to conduct a pragmatic clinical trial to determine the unique contribution of lived experience co‐facilitation to building empathy for voice‐hearers through participation in the HVD simulation workshop. Through this work, we hope to expand the evidence base for the use of lived experience to enhance simulation‐based education for health and social care workers and students.

### 4.2 Relevance to Clinical Practice

People diagnosed with schizophrenia spectrum or other psychotic disorders experience particularly poor outcomes. Empathy is a foundational skill that supports the development of a trusting therapeutic relationship and ultimately contributes to a greater likelihood of recovery for the person receiving care. Our findings indicate that the HVD simulation workshop facilitates the development of empathy for people who hear voices among rural health and social care workers. We advocate that this experiential workshop be offered to rural health and social care workers as part of their training or ongoing professional development. This may be particularly valuable for non‐specialist workers navigating rural environments where workforces and resources may already be stretched. There is also potential to incorporate the workshop into the curriculum for undergraduate psychology, medicine, nursing, and allied health students, with a particular focus on those who are interested in working rurally.

## Author Contributions

C.‐A.S.: designed study and research methodology, co‐facilitated workshops, collected data, contributed to interpretation of data, contributed to writing original manuscript draft, contributed to review and editing of the manuscript. C.M.E.F.: consulted on study design and research methodology, conducted formal quantitative and qualitative analysis and interpretation, contributed to writing original manuscript draft, contributed to review and editing of the manuscript. L.M.: designed study and research methodology, supervised project administration, co‐facilitated workshops, collected data, contributed to interpretation of data, contributed to writing original manuscript draft, contributed to review and editing of the manuscript. All authors agree to be accountable for all aspects of the work.

## Ethics Statement

Ethical approval was obtained from the Human Research Ethics Committee of the University of South Australia (Protocol 204 496).

## Conflicts of Interest

The authors declare no conflicts of interest.

## Supporting information


Table S1.

## Data Availability

The data that support the findings of this study are available on request from the corresponding author. The data are not publicly available due to privacy or ethical restrictions.
