# Supplementary material for: Empathy Increased in Rural and Remote Health and Social Care Workers by Participation in the Hearing Voices That Are Distressing Simulation Workshop
Source: Int J Ment Health Nurs. 2025 Mar 18;34(2):e70027. doi: 10.1111/inm.70027 (PMC11920382; doi:10.1111/inm.70027)
Supplement: Supplementary file 1 — Table S1. [file INM-34-0-s001.docx]

**Supplementary Materials**

Table S1. Modified KCES-R items completed by participants.

| **Section 1: How necessary is it for healthcare professionals to be able to... (1=*Unnecessary* to 7=*Extremely necessary*)** |
| --- |
| 1. Comprehend voice hearers' experiences. 2. Express an understanding of voice hearers’ feelings 3. Values voice hearers’ point of view 4. Consider voice hearers’ feelings to provide consumer-centred care. 5. Be caring in order to build a strong relationship with voice hearers. 6. Identify with voice hearers’ feelings. 7. View the world from the voice hearer’s perspective. |
| **Section 2: I am able to... (1=*Does not describe me* to 7=*Describes me extremely well*)** |
| 1. Comprehend voice hearers’ experiences. 2. Express an understanding of voice hearers’ feelings 3. Value voice hearers’ point of view 4. Consider voice hearers’ feelings to provide consumer-centred care. 5. Be caring in order to build a strong relationship with voice hearers. 6. Identify with voice hearers’ feelings. 7. View the world from the voice hearers’ perspectives. |
